# PLA-HPG based coating enhanced anti-biofilm and wound healing of Shikonin in MRSA-infected burn wound

**DOI:** 10.3389/fbioe.2023.1243525

**Published:** 2023-08-10

**Authors:** Huiyu Han, Lianheng Chen, Shu Liang, Jiawei Lü, Yashi Wu, Xiongjun Wang, Fei Xu, Lanlan Ge, Lingyun Xiao

**Affiliations:** ^1^ Precise Genome Engineering Center, School of Life Sciences, Guangzhou University, Guangzhou, China; ^2^ Center Lab of Longhua Branch, Department of Infectious Disease, Shenzhen People’s Hospital, The Second Clinical Medical College, Jinan University, Southern University of Science and Technology, Shenzhen, Guangdong, China; ^3^ School of Pharmaceutical Sciences (Shenzhen), Sun Yat-sen University, Shenzhen, China; ^4^ Department of Plastic Surgery, Naval Medical Center, Naval Medical University, Shanghai, China

**Keywords:** PLA-HPG, bioadhesive nanoparticles, Shikonin, MRSA biofilm, burn wound

## Abstract

Burn wounds are susceptible to bacterial infections, including Methicillin-resistant *Staphylococcus aureus* (MRSA), which typically form biofilms and exhibit drug resistance. They also have specific feature of abundant exudate, necessitating frequent drug administration. Shikonin (SKN) has been reported to reverse MRSA drug resistance and possesses anti-biofilm and wound healing properties, however, it suffers from drawbacks of low solubility and instability. In this study, we developed PLA-HPG based bioadhesive nanoparticles SKN/BNP, which demonstrated a drug loading capacity of about 3.6%, and exhibited sustained-release behavior of SKN. The aldehyde groups present on the surface of BNP improved the local adhesion of SKN/BNP both *in vitro* and *in vivo*, thereby reducing the frequency of drug dosing in exudate-rich burn wounds. BNP alone enhanced proliferation and migration of the fibroblast, while SKN/BNP promoted fibroblast proliferation and migration as well as angiogenesis. Due to its bioadhesive property, BNP directly interacted with biofilm and enhanced the efficacy of SKN against MRSA biofilm *in vitro*. In a mouse model of MRSA-infected burn wounds, SKN/BNP demonstrated improved anti-biofilm and wound healing efficiency. Overall, our findings suggest that SKN/BNP holds great promise as a novel and effective treatment option for clinical applications in MRSA-infected burn wounds.

## 1 Introduction

Burn is an injury of the skin integrity and subcutaneous tissues, which is generally caused by heat (flames, fluids, solid objects), fire, electricity, chemicals, and radiation (Jeschke et al., 2020). Despite causing considerable pain and suffering, severe burn usually requires hospitalization and can be fatal (Jeschke et al., 2020). Compared to other types of wounds, burns have specific features such as more nonviable tissue, abundant exudate, susceptibility to infection, and prone to scarring (Wang et al., 2018). Due to heat and damage in burn wounds, the permeability of capillaries increases, leading to the leakage of fluid from the capillaries. The duration of exudate from a burn wound varies depending on the severity and depth of the burn, ranging from a few days in first-degree and superficial second-degree burns, to several weeks in deep second-degree and third-degree burns (Jeschke et al., 2020). While the wound bed in burns is covered with a significant amount of exudate and dead tissue, it potentially serves as a natural breeding ground for pathogenic bacteria (Wang et al., 2018). Thus, infections are the most common and potentially serious complications, resulting in delayed healing, additional scar tissue formation, or even sepsis and mortality (Wang et al., 2018). Methicillin-resistant *Staphylococcus aureus* (MRSA) is an antibiotic-resistant colonizer commonly encountered in clinical settings of burn injuries with high morbidity and mortality globally. In addition, infection with *S. aureus* strains may in turn increase the volume of exudate, thereby further complicating the healing process (Vowden et al., 2015). They usually form biofilms at the infection site, which act as an efficient barrier against antimicrobial agents and host immunity, leading to the development of multi-drug resistance and persistent infections. Typically, bacteria embedded in biofilm are up to 1000-fold more resistant to antibiotics compared to planktonic cells of the same strain (Razdan et al., 2022). The pressing challenges underscore an urgent necessity for the discovery of therapeutic alternatives beyond traditional antibiotics to manage MRSA and MRSA biofilm related infections.

Currently, natural products present a potential source of antibacterial agents. *Lithospermum erythrorhizon* (*L. erythrorhizon*), also known as “Zicao,” is a traditional Chinese medicine with a long history to treat infections, inflammation, and hemorrhagic diseases (Shu et al., 2022). Shikonin (SKN), a naphthoquinone compound, is one of the main active components isolated from *L. erythrorhizon* (Kaur et al., 2022a). SKN showed the ability to reverse the drug resistance of MRSA and its clinical strains, and interfere with the formation of *S. aureus* biofilms (Li et al., 2022a). It also possesses many other biological activities, such as anti-inflammatory, wound healing activity, anti-tumor, etc., (Fan et al., 2018; Boulos et al., 2019; Guo et al., 2019). For instance, SKN has been reported to promote angiogenesis (Sakaguchi et al., 2001) and stimulate the proliferation and migration of fibroblasts (Imai et al., 2019), which are key factors influencing the healing of burn wounds. However, SKN has drawbacks of low solubility, short blood circulation time, toxicity or instability, which severely hampered their therapeutic applications (Kaur et al., 2022b). Traditional or novel formulations, involving nanoparticles, could potentially address these limitations. Alkannins and SKN (A/S) based ointment (Helixderm) increased tissue perfusion, collagen production, and epithelial thickness score on second intention wound healing, but it has drawbacks of discomfort, irritation, stickiness and difficulty in cleaning (Karayannopoulou et al., 2011). Drug delivery systems of using nanocarriers in aqueous solution aim at improving the bioavailability of SKN, for example, a SKN liposome possesses high stability of SKN and facilitates a sustained-release pattern, however, in infected burn wounds it needed to be applied topically twice daily (Shu et al., 2022). A SKN formulation with longer retention, as well as non-toxic, biodegradable, biocompatible, is required to reduce the dosing frequency in burn wound therapy.

More frequent dosing or dressing changes are required if massive exudate forms on the wound (Cambiaso-Daniel et al., 2018; Zhou et al., 2022). The concept of bioadhesion—referring to the interaction between polymers, such as nanoparticles, and a biological substrate—offers a promising solution to enhance the duration of drug retention. Polylactic acid—hyperbranched polyglycerol (PLA-HPG) copolymers serve as an optimal nano-carrier for hydrophobic drugs, often referred to as non-bioadhesive nanoparticles (NNP). They exhibit extended circulation in blood due to superior hydrophilicity of the HPG coating. Moreover, when the surface vicinal diols of NNP are oxidated into aldehydes with NaIO_4_ treatment, the newly formed nanoparticles gain tissue adhesion ability via Schiff-base linkages between their surface aldehydes and protein amines, and are thus named bioadhesive nanoparticles (BNP). With topical application, BNP were able to enhance particle uptake and prolong drug retention within limited area, thus limiting systemic drug exposure and side effect (Yu et al., 2022). For example, a sunblock based on padimate O/BNP achieved similar efficacy to commercial sunscreen, but it showed much longer adherence to the stratum corneum and was waterproof (Deng et al., 2015), which reminded us of the application of BNP in exudate-rich burn wounds.

We hypothesized that BNP is a promising drug delivery system, potentially enhancing the solubility and retention of SKN, thereby increasing therapeutic efficiency and reducing dosing frequency in burn wounds. Thus, the objectives of this study included: 1) the development of SKN/BNP to aid in formulating SKN with adhesive and sustained-release effects; 2) exploration of its activity against MRSA *in vitro*; 3) evaluation of the effect of SKN/BNP on fibroblasts proliferation and migration, as well as on angiogenesis; 4) validation of its therapeutic effects in MRSA-infected burn wounds in a mice model.

## 2 Materials and methods

### 2.1 Preparation of SKN/BNP

HPG, PLA-HPG and PLA-cy5 was synthesized as previously described (Deng et al., 2014; Lin et al., 2022). 100 mg PLA-HPG with or without 5 mg SKN were dissolved in 1.2 ml DMSO. The mixture was added to 40 ml deionized water using a syringe under vortex, in order to construct PLA-HPG nanoparticles (NNP). The NNP were filtered with Amico ultra-centrifuge filtration unit (100 kDa) and washed twice with deionized water. 1 vol NNP (resuspended at 25 mg/ml) was incubated with 1 vol of 0.1 M NaIO_4_ for 2 min to obtain BNP, and added with 3 vol of 0.2 M ethylene glycol to quench the reaction. BNP were washed with deionized water and filtered four times with Amico ultra-centrifuge filtration unit (100 kDa), then resuspend at an appropriate concentration.

### 2.2 Characteristics of SKN/BNP

Suspensions of BNP or SKN/BNP were loaded on carbon-supported copper grids and air dried at room temperature. The samples were then stained by 2% phosphotungstic acid and processed to image using a transmission electron microscope [TEM, JEM-2010(Hr), JEOL]. The diameter and polydispersity index (PDI) of BNP and PTX/BNP were determined using a 90Plus PALS particle size analyzer.

For SKN release from SKN/BNP *in vitro*, 1.6 mg SKN in SKN/BNP were loaded in a dialysis bag (MWCO 3.5 kD) and immersed in 80 ml PBS (pH7.4) supplemented with 1% SDS at 37°C in a shaker. The medium was collected at a series of time points, and the amount of SKN released was quantified using an UV-Vis spectrophotometer.

### 2.3 The adhesion assays

SKN or SKN/BNP suspended in PBS (1.5 mg/ml) were added on a lysine-coated glass slide and incubated at 37°C for 30 min. After incubation, the slide was washed five times with PBS, and visualized. *Ex vivo* retention was evaluated using fresh pig skin with the same procedure.

The adhesion of BNP on bacteria biofilm was performed using PLA-cy5 loaded NNP or BNP. In brief, PLA-cy5/NNP and PLA-cy5/BNP were prepared as described (Lin et al., 2022). After biofilm formation, the PLA-cy5/NNP and PLA-cy5/BNP were added to the medium at a final concentration of 1 mg/ml, and incubated at 37°C for 1 h. Then the biofilm was washed three times with PBS and imaged using a fluorescent microscope.

For *in vivo* retention, SKN or SKN/BNP solution (1.5 mg/ml) were added to the wound of anesthetized C57BL/6 mice on day 3 of burn would modeling, when the wound was filled with abundant exudate. After incubation for 2 h, PBS-soaked cotton buds were used to remove non-adherent drugs. Retention of SKN or SKN/BNP on the humid wound were photographed.

### 2.4 Cell proliferation

Murine fibroblast L929 cells were obtained from Procell (Wuhan, China), and were cultured in DMEM supplemented with 10% fetal bovine serum (FBS), 1% penicillin/streptomycin. Cell proliferation was quantified with a Cell Counting Kit-8 (CCK-8) reagent. L929 cells were seeded into a 96-well plate at the density of 5 × 10^3^ cells per well and incubated for 24 h. The cells were then treated with different concentrations of SKN, BNP, SKN/BNP using DMSO as a solvent control for 24 h. After incubation, the supernatant was aspirated and premixed CCK-8 (10 μl) with medium were added to each well, and incubated for 1–3 h. Absorbance was determined at 450 nm.

### 2.5 Wound healing and migration

Wound healing assay was performed using L929 cells and culture inserts (Ibidi) on a 24-well plate. Cells were suspended at 1.4 × 10^6^ cells/ml in DMEM supplemented with 10% FBS, and 70 μL of the cell suspension was transferred to each well of the culture-insert. After 12 h of incubation, the culture-inserts were removed, and the cells were washed thrice with PBS, and treated with SKN, BNP, SKN/BNP using DMSO as a solvent control in DMEM supplemented with 1% FBS. Photos were taken and the gap closure was measured to calculate the migration ability.

### 2.6 Tube formation

The Matrigel tube formation assay using human endothelial EA. hy926 cells was performed to assess *in vitro* angiogenesis (Li et al., 2022b). The EA. hy926 cells were obtained from Procell (Wuhan, China), and were cultured in DMEM supplemented with 10% fetal bovine serum (FBS), 1% penicillin/streptomycin. 250 μl BD Matrigel was pipetted into a well of a 24-well plate and incubated for 1 h at 37°C to coagulate. Then, serum-starved 7.5 × 10^4^ EA. hy926 cells were added to each well and incubated in serum-free medium with SKN, BNP, SKN/BNP using DMSO as a solvent control. After 10 h, capillary-like tubes in each well were photographed with an inverted microscope. The branch points and capillary length were analyzed by ImageJ software.

### 2.7 *In vitro* antibacterial assay

*S. aureus* MRSA (ATCC 43300) was purchased from the American Type Culture Collection (Manassas, VA, United States). It was cultured in 5 ml of Tryptic Soy Broth (TSB) media at 37°C overnight. The bacteria from overnight culture (2% inoculums) were transferred to 10 ml of fresh TSB, and treated with SKN, BNP, SKN/BNP respectively using DMSO as a solvent control. The mixture was incubated at 37°C with shaking at 220 rpm. To monitor the growth trend, samples were withdrawn aseptically at 3-h intervals and OD_600_ of the bacteria was measured to determine bacterial growth kinetics.

The antibacterial activity was also evaluated by measuring the diameter of inhibition zones. The MRSA suspension at 10^6^ cfu/ml was inoculated onto the LB agar media and spread consistently on the plates. 8 mm diameter wells were created on the plates, and 60 µl PBS containing SKN, BNP or SKN/BNP was placed into the wells. After incubation at 37°C for 20 h, the antibacterial activity was determined by measuring the diameter of the inhibition zone.

### 2.8 Quantification of biofilm biomass and viability *in vitro*

Crystal violet was used to quantify biofilm biomass. The initial bacteria concentrations were set according to OD, which had been correlated to a standard curve of OD-cfu. The bacteria were seeded in a 96-well-plate (5 × 10^6^ cfu/well) in TSB medium supplemented with 2% glucose (w/v) and 2% sodium chloride (w/v), and treated with SKN, BNP, SKN/BNP using DMSO as a solvent control. The plate was incubated at 37°C for 24 h to establish biofilm. Then the medium with suspended bacteria were removed by pipetting, followed by washing with PBS twice. The remaining biofilm was stained with crystal violet staining solution for 5 min. Afterwards, the wells were washed twice with DI water, and 33% of acetic acid was added to fully dissolve the crystal violet. The absorbance was measured at 595 nm to determine the biofilm biomass.

For biofilm viability assay, the bacteria were seeded in a 24-well-plate (2 × 10^6^ cfu/well) in TSB medium supplemented 2% glucose and 2% sodium chloride, and treated with SKN, BNP, SKN/BNP as described above for 24 h. Afterwards, all wells were washed 3 times with PBS. The remaining biofilm was dispersed in 1 ml PBS by repeated pipetting and sonication for 30 min. Dispersed biofilm suspension was diluted in PBS, and 25 μl of each dilution was spread on LB agar plate, incubated at 37°C for 16 h. Colonies per plate were counted to determine the number of viable bacteria in the biofilm.

### 2.9 *In vivo* MRSA-infected burn wound healing

The animal study was approved by the Ethics Committee of Guangzhou University. The study was conducted in accordance with the local legislation and institutional requirements. 7-week-old female C57BL/6 mice were housed at room temperature (25°C) and in a light-dark cycle of 12 h. During the experiment, we used 1% pentobarbital sodium (50 mg/kg) to anesthetize the mice via intraperitoneal injection. After anesthetization and shave, a round cutaneous burn wound (φ 12 mm) was created on the back of each mouse, and the wounds were covered with a sterile plastic film. The mice were divided into 4 groups (*n* = 7 or 8 mice per group) and treated with PBS, SKN (30 μg/ml), SKN/BNP (containing 30 μg/ml SKN), BNP (equivalent dose of BNP, 0.83 mg/ml), respectively, on day 1, 5, 9 post-modeling. 1 × 10^7^ cfu of MRSA in PBS were inoculated on the wounds on day 2. After 3 days, obvious yellowish biofilm was seen on the wound infected with MRSA. The wound was dosed and photographed every 4 days. At the end of all procedures (15 days post-modeling), mice were euthanized using the cervical dislocation technique under anesthetization, and tissue samples were harvested. The wound skin was collected and fixed in 4% paraformaldehyde for 24 h, dehydrated, embedded in paraffin. For hematoxylin and eosin (H&E) and Masson’s trichrome staining, tissues embedded in paraffin were cut into 6 and 4 µm sections respectively, and stained following standard procedures. For immunohistochemical staining of CD31, the samples were cut into 4 μm slices. After deparaffinization, the sections underwent antigen retrieval in sodium citrate buffer (pH 6.0) using the high-pressure method. The slices were then incubated with 3% H_2_O_2_ for 15 min to remove the activity of endogenous peroxidase. After blocking with goat serum for 15 min, the slices were incubated with anti-CD31 primary antibody (Cell signaling technology, #77699T, 1:200) at 4°C overnight. The slices were then incubated with HRP-labeled secondary antibody at room temperature for 60 min, and visualized with DAB solution. The slices were counterstained with hematoxylin and analyzed under a microscope.

### 2.10 Statistic analysis

Data were shown as mean ± standard deviation. The statistical trend was analyzed by Student’s *t*-test and was considered as significant if *p* < 0.05.

## 3 Results

### 3.1 Characterization and *in vitro* evaluation of SKN/BNP

**FIGURE 1:**
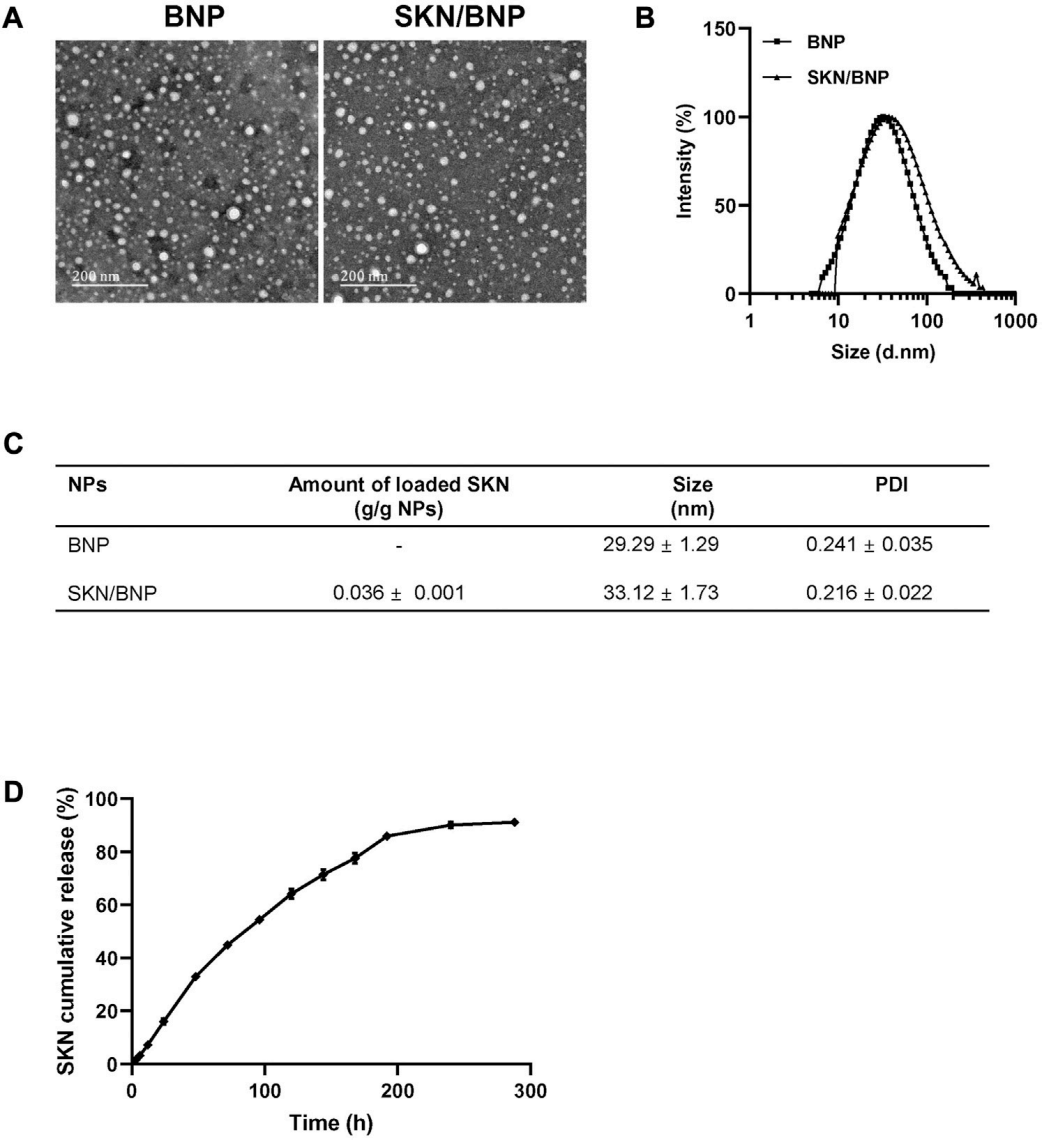
*In vitro* characterization of SKN/BNP. **(A)** TEM images of blank BNP, and SKN/BNP (scale bars, 200 nm). **(B)** Diameters of nanoparticles measured by dynamic light scattering. **(C)** A table showing diameter, PDI, and SKN loading capacity. **(D)** *In vitro* release profile of SKN in PBS supplemented with 1% SDS.

As an amphiphilic copolymer, PLA-HPG self-assembled into nanoparticles (NNP) via a precipitation method in aqueous solution. Using the co-precipitation method, SKN could be incorporated into the hydrophobic core of the nanoparticles (SKN/NNP). The oxidative conversion of NNP to BNP was achieved by NaIO_4_ treatment, which was reported to have no effect on the particle size and appearance of nanoparticles (Mai et al., 2022). Transmission electronic microscopy (TEM) confirmed the spherical shape of the BNP and SKN/BNP (Figure 1A). The diameters of BNP and SKN/BNP were about 29 and 33 nm, respectively, measured by dynamic light scattering (Figures 1B, C), with the average diameter of SK/BNP slightly larger than blank BNP. The polydispersity index of BNP and SKN/BNP was lower than 0.3, indicating the uniformity of the nanoparticles. The amount of SKN loaded in BNP was 3.6% ± 0.1% (g/g nanoparticles). As shown in Figure 1D, SKN displayed a steady and linear release profile. There was only 16% SKN released from SKN/BNP during the initial 24 h of incubation without burst release, and the rest of the encapsulated drug (∼85%) was slowly released over a period of 8 days in PBS supplemented with 1% SDS at 37°C. These results showed that BNP could be used as a drug delivery system for continuous and sustained drug release for SKN.

### 3.2 BNP improved local adhesion of SKN *in vitro* and *in vivo*

**FIGURE 2:**
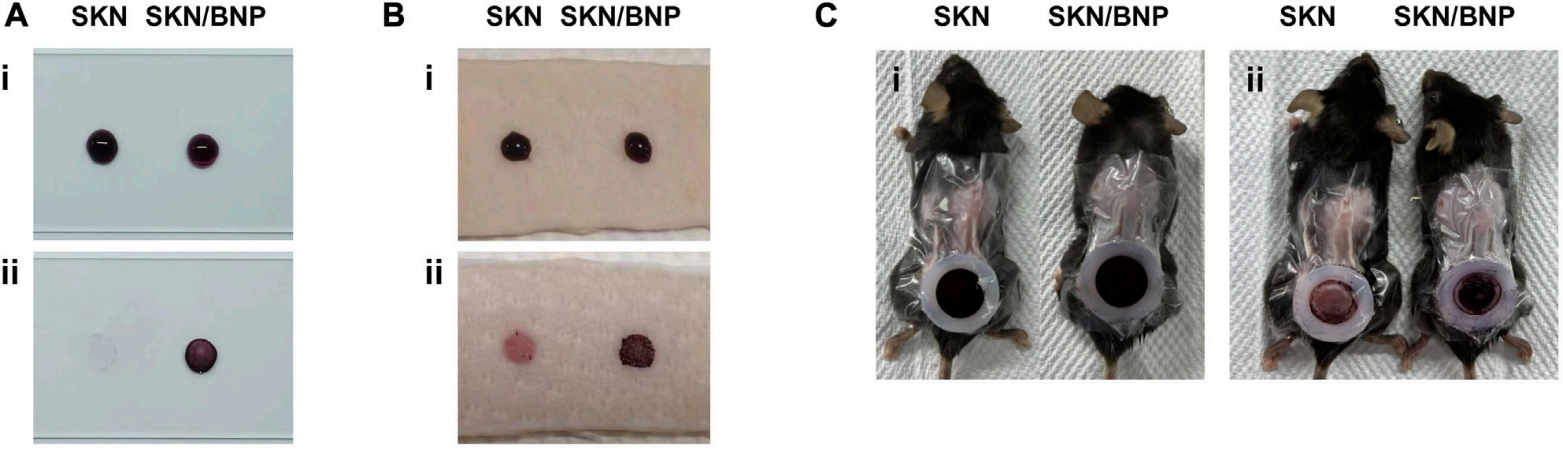
Evaluation of SKN/BNP adhesion. Retention of SKN and SKN/BNP on **(A)** polylysine-coated slides, **(B)** pig skin, and **(C)** burn wound in mice. SKN and SKN/BNP at 1.5 mg/ml were applied respectively, followed by incubation at 32°C for 0.5 h (for polylysine-coated slides and pig skin) or 2 h (for burn wound). After washing with PBS thoroughly, the retention was imaged.

The aldehyde groups on the surface of BNP could form Schiff-base bonds with the amino groups on substances such as polylysine and proteins, resulting biological adhesion. To test whether BNP provides prolonged retention for SKN, polylysine-coated slides and pig skin were incubated with 1.5 mg/ml SKN or SKN/BNP at 32°C for 0.5 h. SKN/BNP showed rapid and robust adhesion compared with free SKN (Figures 2A, B). In addition, the adhesive effectiveness of SKN/BNP on burn wound with abundant exudate 3 days after modeling was also tested. Similarly, SKN/BNP at 1.5 mg/ml also adhered strongly to the burn wound after 2-h incubation at 32°C (Figure 2C), and even gentle wiping with PBS-soaked cotton buds can hardly remove the purple substance of SKN/BNP.

### 3.3 SKN/BNP enhanced proliferation and migration of fibroblast, as well as promoted angiogenesis

**FIGURE 3:**
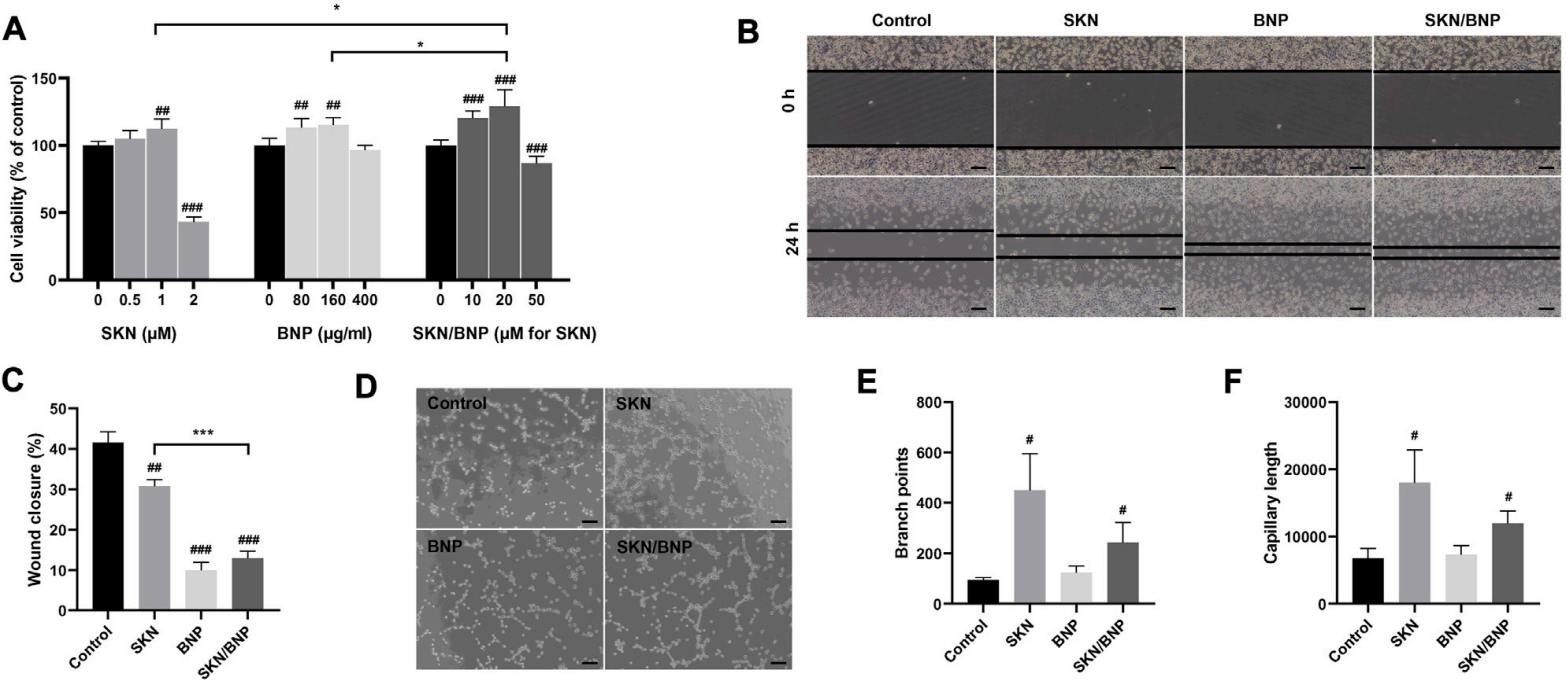
The effect of BNP and SKN/BNP on proliferation, migration and angiogenesis. **(A)** Cell viability of L929 after treatment of SKN (0.5, 1, and 2 μM), SKN/BNP (10, 20, and 50 μM) and equivalent dose of BNP (80, 160, and 400 μg/ml) for 24 h. **(B)** Effects of SKN, BNP, and SKN/BNP on L929 migration and **(C)** the wound closure distance was quantified. Magnification, ×100, scale bars, 200 μm. **(D)** Effects of SKN, BNP, and SKN/BNP on promoting angiogenesis of EA.hy926, and **(E)** branch points and **(F)** capillary length were measured using ImageJ. Magnification, ×100, scale bars, 200 μm. The L929 and EA.hy926 cells were treated with 1 μM SKN, 160 μg/ml BNP, and 20 μM SKN/BNP. ^#^ *p* < 0.05; ^##^ *p* < 0.01; ^###^ *p* < 0.001, compared with the control group. *p < 0.05; ***p < 0.001.

The healing of burns requires fibroblast proliferation, which serves a vital role in filling the wound and synthesizing collagen fibers involved in wound healing (Bi et al., 2020). As only ∼16% of SKN were released from SKN/BNP after 24 h of incubation (Figure 1D), SKN/BNP at higher dose of free SKN was used for comparative studies. SKN at 1 μM increased cell viability of L929 (*p* < 0.01, Figure 3A), however, SKN at 2 μM inhibited cell growth significantly (*p* < 0.001), demonstrating that SKN may induce cytotoxicity when applied at high concentration. Interestingly, BNP at the concentration of 80 and 160 μg/ml also promoted cell proliferation of L929 cells (*p* < 0.01), and with increasing concentrations up to 400 μg/ml, it had no influence on cell viability compared with the control group (Figure 3A). BNP at high concentrations (1 and 1.5 mg/ml) also had no toxicity in L929 cells (Supplementary Figure S1). Other studies also reported that unloaded BNP are non-toxic and did not show any toxicity at 1 mg/ml (Deng et al., 2016). SKN/BNP promoted cell proliferation in a dose-dependent manner, and its greatest effect at 20 μM was stronger than that of SKN or BNP alone (Figure 3A). In addition, 1 μM SKN, 20 μM SKN/BNP and equivalent dose of BNP (160 μg/ml) promoted the cell migration of L929 fibroblasts (*p* < 0.01, Figures 3B, C).

Angiogenesis is another crucial process involved in wound healing (Li et al., 2022b). To further determine their effect in angiogenesis, we investigated the tube-forming ability of EA.hy929 cells. Our results suggested both SKN (1 μM) and SKN/BNP (20 μM) possessed the ability to stimulate tube formation, as indicated by increased branch points and capillary length (*p* < 0.05, Figures 3D–F). We also noticed that the effect of SKN/BNP showed a slight lower trend than free SKN, which might be caused by less SKN released during shorter incubation time of 10 h.

### 3.4 BNP directly interacted with biofilm and amplified the effect of SKN against MRSA biofilm

**FIGURE 4:**
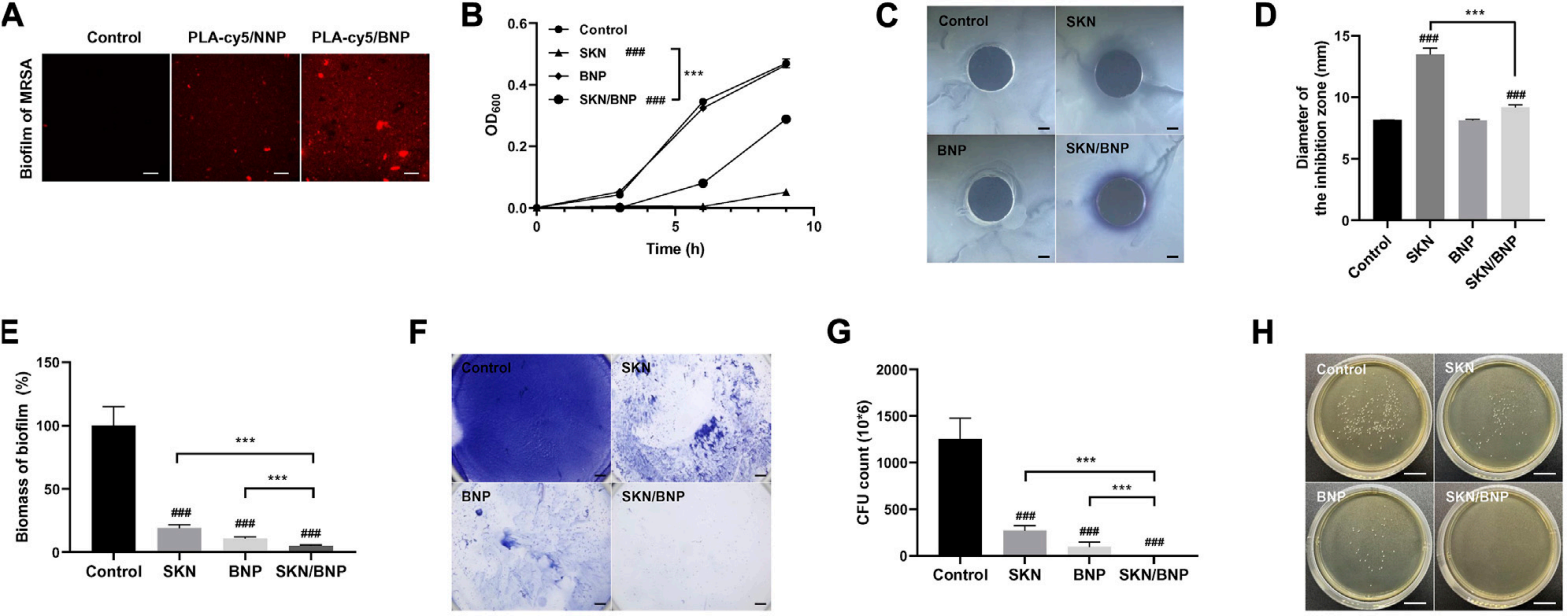
The effect of SKN/BNP on the growth and biofilm formation of MRSA. **(A)** Fluorescent images of residual PLA-cy5 on MRSA biofilm, PLA-cy5/NNP and PLA-cy5/BNP at 1 mg/ml were incubated with MRSA biofilm at 37°C for 1 h. Scale bars, 20 μm. **(B)** Bacterial growth curves of MRSA. **(C,D)** The effect of 20 μM SKN, 3.2 mg/ml BNP, and 400 μM SKN/BNP on the inhibition zone of MRSA. Scale bars, 2,000 μm. **(E,F)** The biomass of residual MRSA biofilm. The biofilm was stained with crystal violet. Scale bars, 2,000 μm. **(G)** The number of live bacteria in remaining MRSA biofilm. **(H)** Representative photographs of MRSA colonies. Scale bars, 2 cm. ^###^ *p* < 0.001, compared with the control group. ***p < 0.001.

The converting of HPG on the surface of NNP to an aldehyde-rich corona grants BNP an unprecedented bioadhesive property in the skin tissue. The adhesion of BNP on biofilm has not been investigated. 1 mg/ml PLA-cy5/NNP or PLA-cy5/BNP was incubated with mature MRSA biofilm for 1 h, and free nanoparticles were removed via washing with PBS. PLA-cy5/BNP showed substantially higher biofilm retention than PLA-cy5/NNP (Figure 4A), indicating BNP might be an ideal carrier for topical antimicrobial drug delivery in combating biofilm.

In general, SKN has been reported by to be a potent phytomedicine against gram-positive bacteria, including *S. aureus*, as well as against various species of lactic acid bacteria (Andújar et al., 2013). As shown in Figure 4B, SKN at 20 μM blocked the growth of MRSA over a 9-h period (*p* < 0.001), while SKN/BNP at 400 μM inhibited MRSA growth to a lesser extent (*p* < 0.001). BNP alone had little effect on the proliferation of MRSA (Figure 4B). Similar results were seen in terms of inhibition zone diameter (Figures 4C, D). BNP did not show any detectable inhibition zone, while 20 μM SKN had larger inhibition zone than 400 μM SKN/BNP (*p* < 0.001). This may be induced by the lower concentration of SKN released from the SKN/BNP during incubation. In addition, SKN/BNP at 400–1,200 μM showed progressive dose-dependent inhibitory activity against MRSA both in bacterial growth kinetics and diameter of the inhibitory zone (*p* < 0.001, Supplementary Figures S2A–C).

Crystal violet staining revealed that 20 μM SKN significantly inhibited the formation of MRSA biofilm (*p* < 0.001, Figures 4E, F), as reported previously (Lee et al., 2015). Surprisingly, BNP also had a significant effect on the biomass of residual MRSA biofilm (*p* < 0.001). In the experimental flow of biofilm staining using crystal violet, we noticed that the amount of biofilm remained almost the same before washing with PBS, however, it broke into pieces with a gentle shake and it could be easily removed from the plate. These phenomena indicated that BNP might affect the structural integrity of MRSA biofilm. SKN/BNP showed the best efficiency in reducing the biomass of MRSA biofilm (*p* < 0.001, Figures 4E, F). With BNP/SKN retention in the biofilm, it might exert a close-range effect on bacteria within the biofilm and avoid the shielding effect of the biofilm on exogenous free drugs. The cfu count of bacteria in remaining biofilm after different treatment also showed that SKN/BNP is significantly more potent in inhibiting MRSA biofilm than SKN or BNP individually (*p* < 0.001, Figures 4G, H).

### 3.5 SKN/BNP enhanced wound healing in MRSA infectious burn wound *in vivo*

**FIGURE 5:**
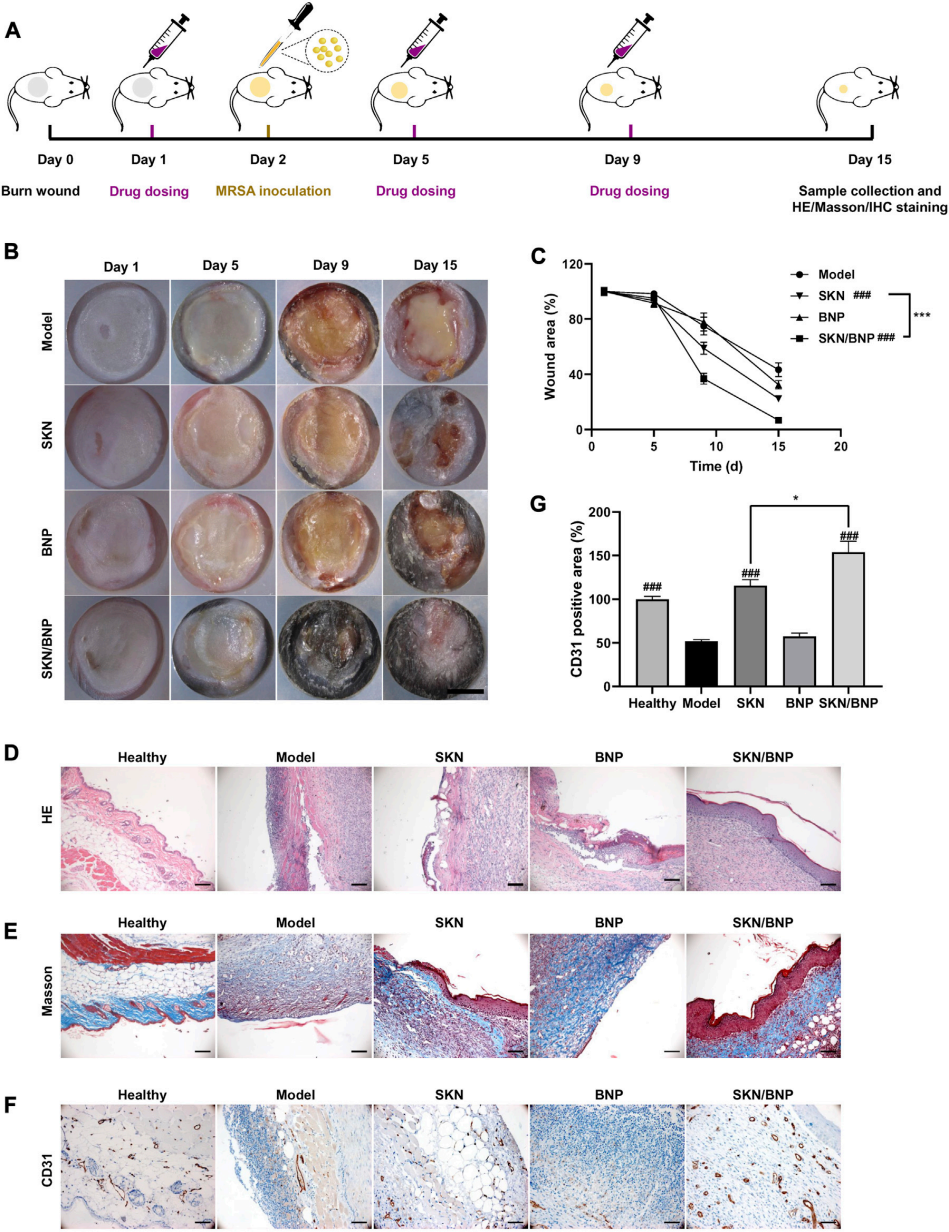
SKN/BNP enhanced MRSA-infected wound healing *in vivo*. **(A)** Experiment flowchart. **(B)** Photographs of MRSA-infected burn wounds after different treatments. Scale bar, 5,000 μm. **(C)** Quantification of unhealed wound area. **(D)** H&E and **(E)** Masson’s trichrome staining images of mice epidermis. **(F)** Immunohistochemical staining images and **(G)** quantification of CD31 expression in burn wounds. Scale bars, 100 μm. ^###^ *p* < 0.001, compared with the model group. *p < 0.05; ***p < 0.001.

`A burn wound with a 12 mm diameter was created on the back of C57BL/6 mice at Day 0. PBS, SKN (30 μg/ml), SKN/BNP (containing 30 μg/ml SKN), BNP (equivalent dose of BNP, 0.83 mg/ml) were applied to the wound on Day 1 post-modeling. Then precultured MRSA was inoculated to establish the mouse model with an infected burn wound (Figure 5A). As showed in Figure 5B, much exudate and obvious yellow biofilm existed on the wound on day 5, the biofilm turned much thicker on day 9. Treatment of SKN and SKN/BNP reduced the formation of MRSA biofilm effectively (*p* < 0.001), whereas BNP had little effect on the amount of biofilm *in vivo* (Figure 5B). Wound size in Figure 5C showed that the area of the burn wound began to reduce at least 5 days post-modeling. It also demonstrated that wound healing was faster in mice treated with SKN and SKN/BNP, with SKN/BNP exhibiting better therapeutic effect than SKN on Day 9 and Day 15 (*p* < 0.05). However, BNP also show little effect on wound size (Figure 5C). Consistently, histopathologic examination of the burn wound by H&E staining on Day 15 showed the existence of biofilm and profuse inflammatory infiltrates in the model group. With the treatment of SKN or SKN/BNP, the thickness of the biofilm significantly reduced, and reepithelialization almost completed in mice treated with SKN/BNP (Figure 5D). Masson staining showed almost none residual cutaneous appendages, severe collagen denaturation in mice of the model group. More collagenous fibers (stained in blue) were found after SKN and SKN/BNP treatment (Figure 5E). Notably, immunohistochemical examination demonstrated the expression of CD31, a specific endothelial marker to detect angiogenesis, were significantly elevated in mice treated with SKN and SKN/BNP, compared with the mice in the model group (*p* < 0.001, Figures 5F, G). The microvessel density was higher in mice treated with SKN/BNP than that treated with SKN (*p* < 0.05), indicating better angiogenesis with the adhesive and sustained release features of SKN from SKN/BNP (Figures 5F, G). Taken together, these results suggested that BNP based coating could enhance the wound healing ability and angiogenesis of SKN in MRSA-infected burn wound.

We assessed the *in vivo* toxicity of SKN, BNP and SKN/BNP using H&E staining. As showed in Supplementary Figure S3, no obvious structural abnormality was observed in the major organ of mice treated with SKN, BNP and SKN/BNP compared with those in the model group, indicating good compatibility of SKN/BNP in anti-infectious and burn wound therapies.

## 4 Discussion

Topical drug application is an appealing option in burn and wound care because it increases site-specific drug concentration and reduced adverse effects of systemic administration. Creams, ointments, gel, or films are the dosage forms used for burns, with dosing frequencies ranging from several times a day to once every one or 2 days (Tiwari et al., 2021). The amount of exudate may interfere with the retention of topical applied drugs, and more frequent dosing is required if massive exudate forms on the wound (ISBI Practice Guidelines Committee, 2016). Gently rubbing may also inadvertently remove topically applied medication, thereby increasing dosing frequency. The frequent dressing changes can lead to discomfort and pain for patient, moreover, they traumatize recently healed surfaces and may impede the healing process for newly formed epithelial tissue (ISBI Practice Guidelines Committee, 2016). Thus, the development of a topical carrier that enables prolonged retention and sustained drug release within burn wounds is of utmost necessity.

Herbal drugs are extremely useful for treating wounds and burn wounds. Different studies have demonstrated the effect of *A. euchroma* in burn or excisional wounds healing (Mohsenikia et al., 2017). Topical *A. euchroma* extract with carboxymethylcellulose (CMC) gels significantly improved wound closure rate, fibroblast population, volume density of collagen bundles, and length density of vessels on third-degree burn wound (Ashkani-Esfahani et al., 2012). *A. euchroma* ointment has benefits over silver sulfadiazine in the treatment of second-degree burn wounds in a randomized clinical trial (Nasiri et al., 2016). SKN is one of the main components of *A. euchroma* that exhibits remarkable pharmacological property of wound healing, as well as anti-fungal, anti-bacteria, anti-virus (Kumar et al., 2021). For example, SKN promotes the proliferation of fibroblasts, collagen fiber levels of the granuloma tissue (Sakaguchi et al., 2001). Furthermore, the advantage of SKN over commonly used antibiotics is their broad-spectrum antibacterial potential, especially against multi-drug-resistant bacterial strains (Lee et al., 2015). SKN has been reported to reduce the growth of six clinical MRSA isolates, and synergize with various membrane-permeabilizing agents, ATPase inhibitors or antibiotics against MRSA (Lee et al., 2015; Li et al., 2022a). These characteristics make SKN particularly suitable for the treatment of infected wounds.

However, the therapeutic application of SKN is limited by its poor solubility and chemical stability in aqueous medium, low oral bioavailability, the need for multiple doses and non-selective high toxicity (Kaur et al., 2022b). Utilizing drug delivery systems can greatly overcome the limitations associated with SKN. Currently, research on drug delivery of SKN mainly focuses on systemic administration, the formulations of which include nanomedicines including liposomes, polymeric micelles, nanoparticles, nanoemulsions, nanogels, chimeric advanced drug delivery nano systems, etc., (Yan et al., 2023). For example, SKN nanoparticles coated with natural surfactants saponin and sophorolipid, increased the encapsulation efficiency and *in vitro* bioavailability of SKN (He et al., 2023). PEGylated liposomes formulated with negatively charged lipid (DSPG) combined with DOPC demonstrated increased incorporation efficiency and stability, reduced particle sizes of SKN, when compared with conventional ones (Tsermentseli et al., 2018). On the contrary, there are few studies related to topical SKN delivering. SKN liposome showed a good sustained-release behavior, yet it needed to be applied topically on the skin twice daily (Cambiaso-Daniel et al., 2018). Self-assembled SKN-Fe(III) nanoparticles (SKN-Fe NPs) imposed dose-dependent anti-inflammatory and antibacterial activity, as well as wound-healing properties, overcoming the inherent limitations of SKN’s low solubility. However, SKN-Fe NPs still had the disadvantage of requiring frequent dosing and needed to be encapsulated in hydrogels when applied topically (Hu et al., 2022). In this study, the PLA-HPG-based coating was found to improve the solubility and stability of SKN in aqueous solution, facilitating sustained release of SKN for over 7 days. More importantly, when the vicinal diols of PLA-HPG nanoparticles were converted into an aldehyde-rich corona by NaIO_4_ treatment, it could form Schiff-base linkages with primary amines on proteins, thus enhancing the adhesion of SKN nanoparticles to biological surfaces including the skin tissues. Furthermore, with the greatest effect of promoting fibroblast proliferation observed at 1 μM, higher concentrations (10 and 100 µM) increased LDH activity and were cytotoxic (Imai et al., 2019). BNP was able to reduce the toxicity of encapsulated drugs, which was proved by a previous study that a sunblock based on padimate O/BNP prevented ROS medicated double-stranded DNA induced by padimate O, while achieving similar efficacy to commercial sunscreen (Deng et al., 2015). Thus, this system is highly effective in reducing the local dosing frequency of SKN, and the local adhesive feature of SKN/BNP may inhibit toxicity or systemic absorption of SKN, thus minimizing side effect.

The adhesion of BNP allows it to insert into the interior of a biofilm during its formation, and might affect the structural integrity of MRSA biofilm. SKN/BNP combines the antibacterial effect of SKN and adhesiveness of BNP, which allows SKN/BNP to remain inside the biofilm and release SKN site-specifically to exerting a close-range inhibitory effect. Thus, BNP and SKN had a synergistic effect against MRSA biofilm formation (Figures 4E–H). This effect bears resemblance to how Epothilone B/BNP inhibits the formation of metastatic foci in peritoneal ovarian cancer (Deng et al., 2016). In an *in vitro* model using poly-lysine-coated glass slides, pre-incubation of Epothilone B/BNP on the slide surface significantly reduced suppressed tumor adhesion and growth compared with free Epothilone B at the same dosage in subsequent incubation with USC cells, even though the cytotoxicity of free Epothilone B is higher than Epothilone B/BNP in equivalent dose. Therefore, by leveraging the bioadhesive property, drug delivery with BNP to the lesion interior might be more effective than free drugs or other nanoparticles.

To our surprise, blank BNP alone promoted the proliferation and migration of fibroblasts at low concentrations (80 and 160 μg/ml), whereas NNP cannot (Supplementary Figure S4A). Culture medium replacement to remove BNP after 8-h incubation cannot eliminate the effect of BNP in promoting cell proliferation (Supplementary Figure S4B). Previous studies have explored the cytotoxicity of NNP and BNP, and found that at a concentration of 1 mg/mL, neither had a cytotoxic effect on various cells, including USC, A549, B16, HEK293, LNCaP, Deng et al. (2016). Consistently, our results demonstrated that blank BNP had no toxicity in L929 at 1 mg/ml, suggesting good biocompatibility of BNP (Supplementary Figure S1), yet it promoted L929 cell growth at lower concentrations (Figure 3A). The mechanism by which BNP promotes cell proliferation has not been investigated, and further research is needed to determine whether it is a common feature of aldehyde nanoparticles or aldehyde-based material. Another possibility is that PLA-HPG itself can affect cell proliferation, and differences in cellular uptake between BNP and NNP may lead to varying impacts on cell growth. NNP, with its hydroxyl-rich surface, acts as a “stealthy” coating with extended circulation time, reducing cellular uptake (Deng et al., 2016). On the other hand, BNP, with its aldehyde-rich surface, binds to the cell surface and might enter cells through phagocytosis (Deng et al., 2016). A recent study has shown that PLA nanoparticles promote the proliferation of senescent fibroblasts by upregulating the PI3K/AKT signaling pathway, which indicates that BNP may also regulate the PI3K/AKT pathway (Oh et al., 2023). Western blot analysis was performed on BNP- and NNP-treated L929 cells, and it revealed that BNP at 80 and 160 μg/ml, or NNP at 160 μg/ml, upregulated the phosphorylation of AKT (Ser472) (Supplementary Figure S5). BNP and NNP had no influence on the phosphorylation of ERK1/2 (Thr202/Tyr204), AMPK (Thr172) and NF-κB (Ser563). However, BNP did modulate the level of phosphorylated c-Jun (Ser73) significantly. Extensive experiments are needed to fully elucidate the mechanisms underlying the promotion of cell proliferation by BNP. Furthermore, at appropriate concentrations SKN/BNP promotes cell proliferation more strongly than BNP or SKN alone, indicating a combined effect between BNP and SKN on cell proliferation. In MRSA-infected wounds, we noticed that the application of blank BNP did not significantly promote wound healing (Figures 5B, C), possibly due to its inability to inhibit MRSA bacterial growth and reduce biofilm formation alone *in vivo*.

## 5 Conclusion

**FIGURE 6:**
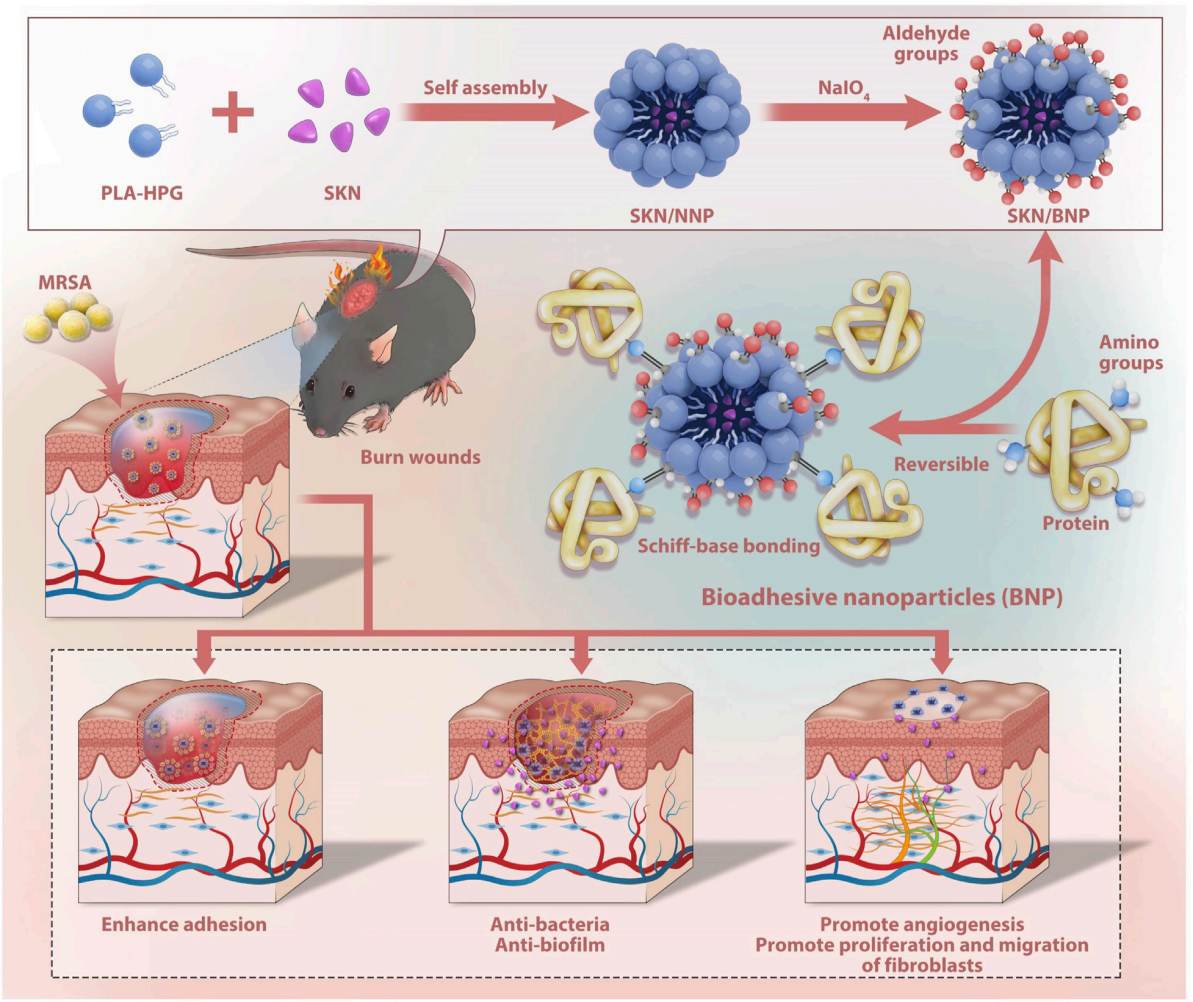
Diagram illustrating the synergistic effect of BNP and SKN assembled as SKN/BNP in promoting burn wound healing.

In summary, SKN/BNP exhibited enhanced solubility and sustained release of SKN, along with long-term retention on exudate-rich burn wounds. Additionally, it promoted the proliferation and migration of fibroblasts, as well as angiogenesis. Furthermore, BNP directly interacted with biofilm, and amplified therapeutic effects of SKN against MRSA biofilm both *in vitro* and *in vivo* (Figure 6). Overall, our findings indicate that SKN/BNP holds great promise as a novel and effective treatment option for clinical applications in MRSA-infected burn wounds.

## Data Availability

The raw data supporting the conclusion of this article will be made available by the authors, without undue reservation.
