# Supplementary material for: PLA-HPG based coating enhanced anti-biofilm and wound healing of Shikonin in MRSA-infected burn wound
Source: Front Bioeng Biotechnol. 2023 Aug 10;11:1243525. doi: 10.3389/fbioe.2023.1243525 (PMC10448828; doi:10.3389/fbioe.2023.1243525)
Supplement: Supplementary file 1 [file DataSheet1.docx]

Supplementary Material

PLA-HPG based coating enhanced anti-biofilm and wound healing of Shikonin in MRSA infected burn wound

Huiyu Han ^1,†^, Lianheng Chen ^1,†^, Shu Liang ^2,3,†^, Jiawei Lü ^1^, Yashi Wu ^1^, Xiongjun Wang ^1^, Fei Xu ^4,^*, Lanlan Ge ^2,^* and Lingyun Xiao ^1,2,^*

*** Correspondence**: Corresponding Author: fei_307@163.com (F. Xu), gelanlan4710@163.com (L. Ge), xiaolingyun@gzhu.edu.cn (L.Y. Xiao)


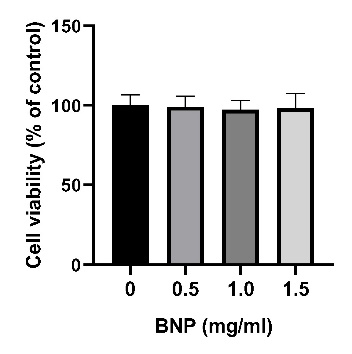


**Supplementary Figure 1.** The effect of 0.5, 1.0, 1.5 mg/ml BNP on the proliferation of L929 cells.


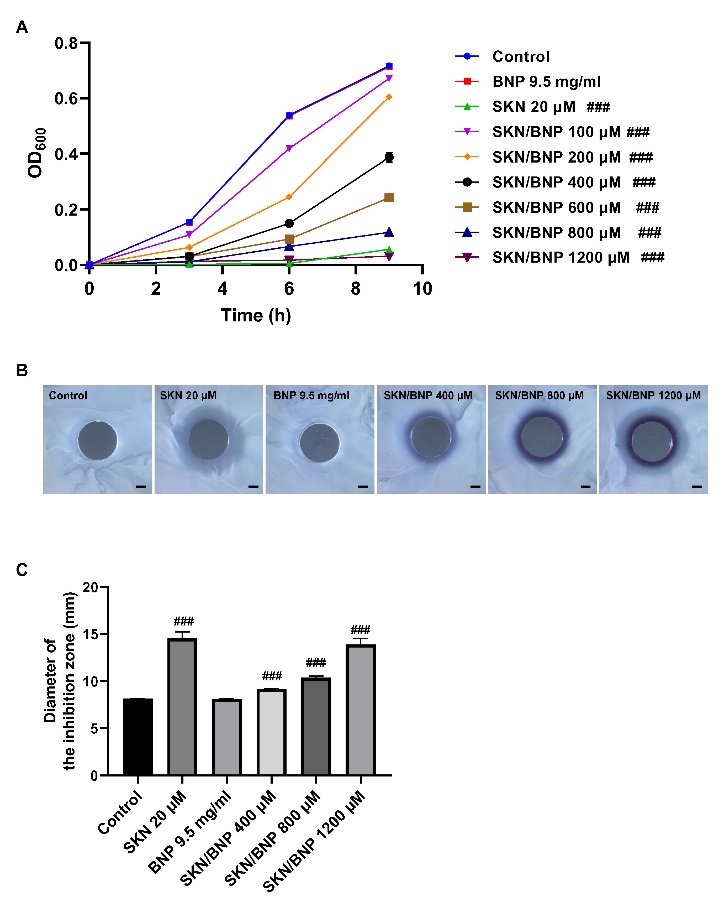


**Supplementary Figure 2.** (A) Bacterial growth curves of MRSA treated with different concentrations of SKN/BNP. (B,C) The effect of 20 μM SKN, 9.5 mg/ml BNP, and different concentrations of SKN/BNP on the inhibition zone of MRSA. Scale bars, 2000 μm. ###, *p* < 0.001, compared with the control group.


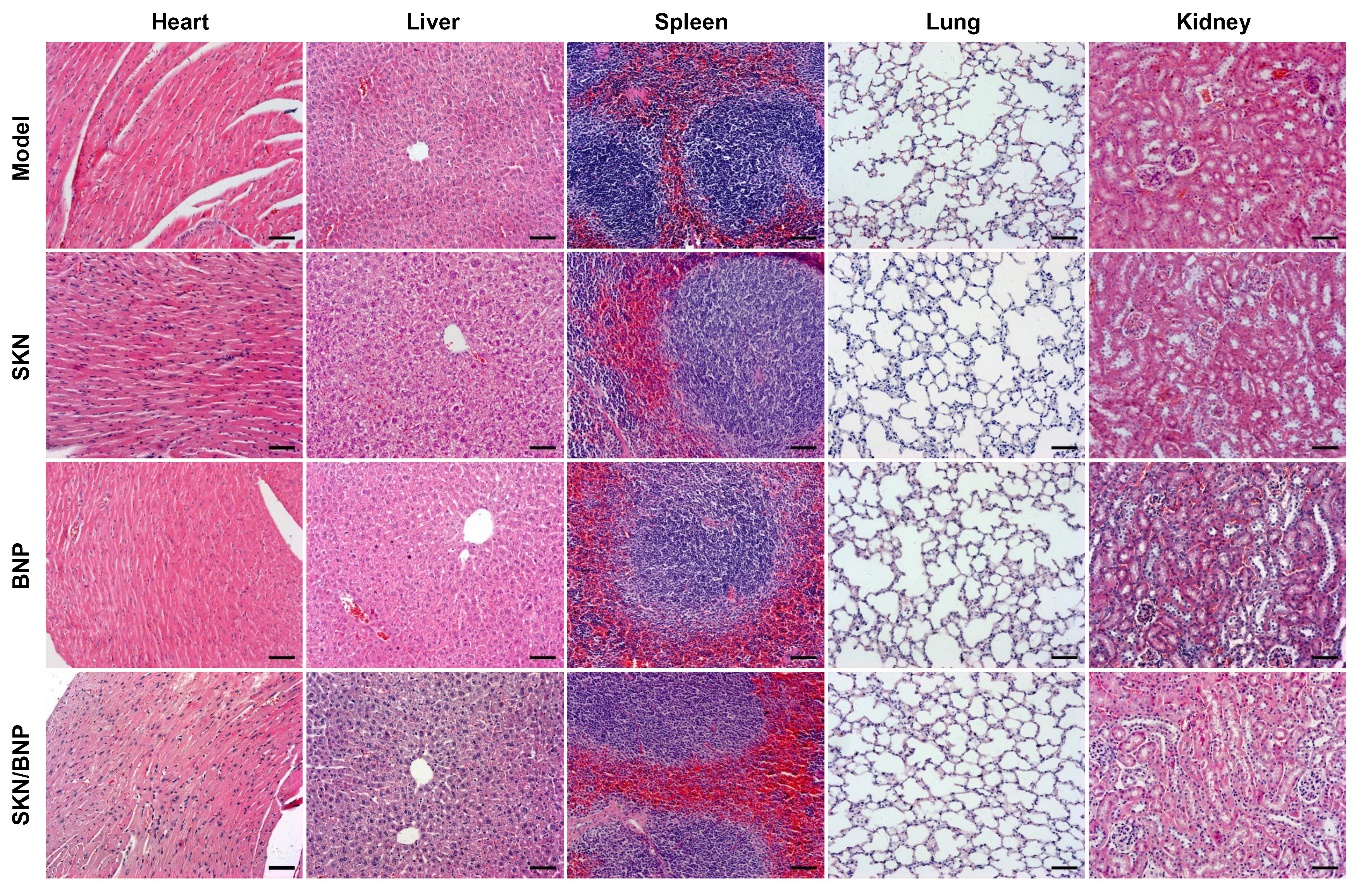


**Supplementary Figure 3.** Histological H&E staining of main organs from mice treated in different groups (scale bars, 100 μm).


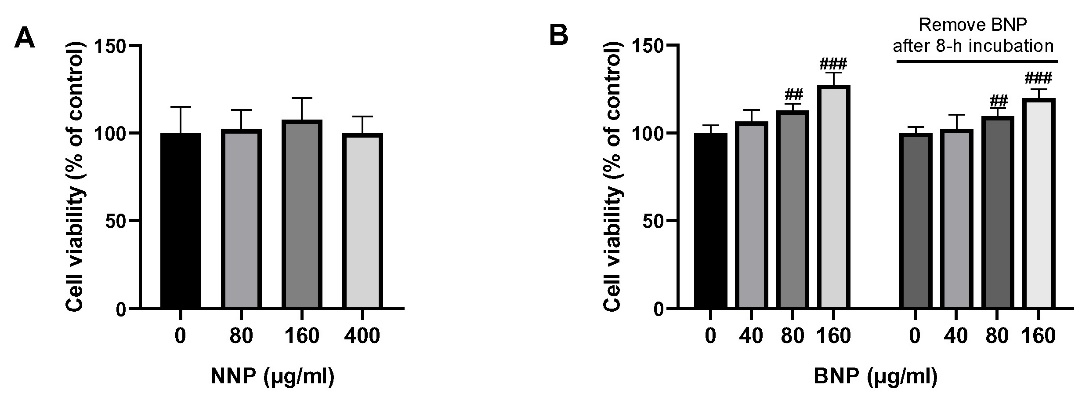


**Supplementary Figure 4.** The effect of NNP and BNP on the proliferation of L929 cells. (A) The cells were treated with 80, 160, 400 μg/ml NNP for 24 h; (B) The cells were treated with 40, 80, 160 μg/ml BNP for 24 h, or incubated for 8 h followed by culture medium replacement to remove BNP and continue cultivation for 16 hours. ##, *p* < 0.01; ###, *p* < 0.001, compared with the control group.


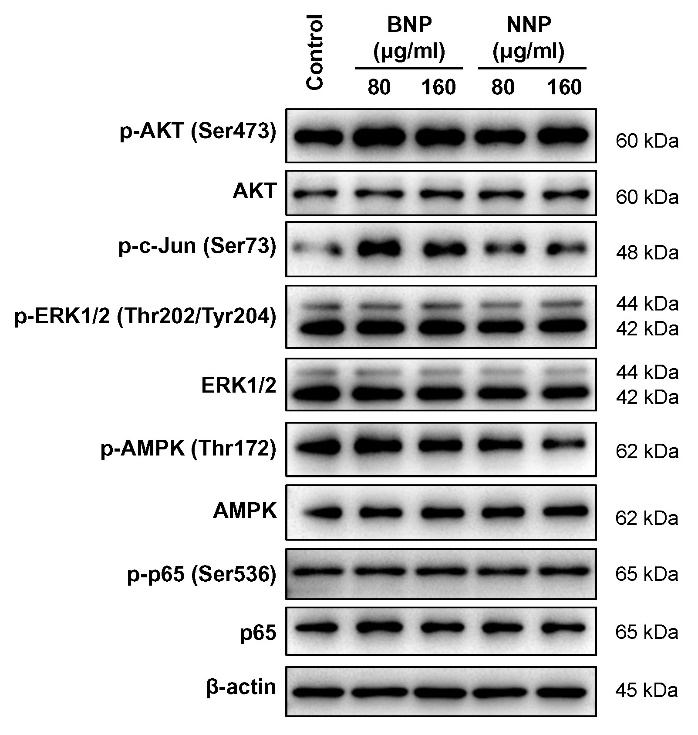


**Supplementary Figure 5.** Western blot analysis of the effect of BNP and NNP on L929 cells.
